# An investigation into the effects of simulated ischaemic preconditioning on mitochondrial fusion in mouse embryonic fibroblasts

**DOI:** 10.1186/1749-8090-10-S1-A64

**Published:** 2015-12-16

**Authors:** Ernest J Chew, Andrew R Hall, Niall F Burke, Derek J Hausenloy, Derek Yellon

**Affiliations:** 1 The Hatter Cardiovascular Institute, University College London, 67 Chenies Mews, London WC1E 6HX, UK

## Background/Introduction

Ischaemic conditioning is the cardioprotective process of exposing the heart to short periods of ischaemia and reperfusion in order to increase its survivability when encountered with a subsequent sustained period of lethal ischaemia. Mitochondria undergo fusion and fission processes and potentiating mitochondrial fission has been reported to be linked to increased cell death.

## Aims/Objectives

Investigate if the beneficial effect of ischaemic pre-conditioning is mediated by mitochondrial fusion.

## Method

Mouse embryonic fibroblasts (MEFs) were divided into three groups: (i) Normoxia, (ii) simulated ischaemia-reperfusion injury (SIRI) and (iii) hypoxic pre-conditioned (Pre-con). Mitochondrial morphology was then determined using confocal imaging and cell death was assessed by flow cytometry.

## Results

Cell death was 2.1% ± 1.4%, 30% ± 13.2% and 10% ± 6.8% in Normoxia, SIRI and Pre-con groups respectively (p < 0.0001). Mitochondria morphology studies showed that MEFs that underwent period of SIRI had a 46% ± 6.1% decrease in fused mitochondria compared to normoxic controls. More importantly, MEFs which have undergone hypoxic pre-conditioning before the period of SIRI showed an increase in the amount of fused mitochondrial networks compared to MEFs which were exposed to SIRI only (19.3% ± 1.6 vs 4.5% ± 6.4% respectively, p < 0.05). However, Hypoxic pre-conditioning does not seem to be inducing mitochondrial fusion as MEFs exposed to the pre-conditioning protocol only, showed a 16% ± 2.1% decrease in mitochondrial fusion.

## Discussion/Conclusion

This study demonstrates that ischaemic pre-conditioning can be replicated in a cell line and that it appears to be preventing cell death by inhibiting mitochondrial fission. Further studies could are needed to investigate the effects of inhibiting different levels of the mitochondrial fission pathway in different cell line and animal models.

